# m7G Methylation-Related Genes as Biomarkers for Predicting Overall Survival Outcomes for Hepatocellular Carcinoma

**DOI:** 10.3389/fbioe.2022.849756

**Published:** 2022-05-10

**Authors:** Xin-Yu Li, Zhi-Jie Zhao, Jing-Bing Wang, Yu-Hao Shao, Hui-Liu, Jian-Xiong You, Xi-Tao Yang

**Affiliations:** ^1^ Department of Interventional Therapy, Shanghai Ninth People’s Hospital, Shanghai Jiao Tong University School of Medicine, Shanghai, China; ^2^ Department of Neurosurgery, Shanghai Ninth People’s Hospital, Shanghai JiaoTong University School of Medicine, Shanghai, China; ^3^ Department of Ophthalmology, Shanghai Tenth People’s Hospital, Tongji University, Shanghai, China; ^4^ Department of Nephrology, Shanghai Jiao Tong University Affiliated Sixth People’s Hospital, Shanghai, China

**Keywords:** hepatocellular carcinoma, N7-methyladenosine, prognosis, model, HCC

## Abstract

**Aim:** The search for prognostic biomarkers and the construction of a prognostic risk model for hepatocellular carcinoma (HCC) based on N7-methyladenosine (m7G) methylation regulators.

**Methods:** HCC transcriptomic data and clinical data were obtained from The Cancer Genome Atlas database and Shanghai Ninth People’s Hospital, respectively. m7G methylation regulators were extracted, differential expression analysis was performed using the R software “limma” package, and one-way Cox regression analysis was used to screen for prognostic associations of m7G regulators. Using multi-factor Cox regression analysis, a prognostic risk model for HCC was constructed. Each patient’s risk score was calculated using the model, and patients were divided into high- and low-risk groups according to the median risk score. Cox regression analysis was used to verify the validity of the model in the prognostic assessment of HCC in conjunction with clinicopathological characteristics.

**Results:** The prognostic model was built using the seven genes, namely, CYFIP1, EIF4E2, EIF4G3, GEMIN5, NCBP2, NUDT10, and WDR4. The Kaplan–Meier survival analysis showed poorer 5-years overall survival in the high-risk group compared with the low-risk group, and the receiver-operating characteristic (ROC) curve suggested good model prediction (area under the curve AUC = 0.775, 0.820, and 0.839 at 1, 3, and 5 years). The Cox regression analysis included model risk scores and clinicopathological characteristics, and the results showed that a high-risk score was the only independent risk factor for the prognosis of patients with HCC.

**Conclusions:** The developed bioinformatics-based prognostic risk model for HCC was found to have good predictive power.

## Introduction

Hepatocellular carcinoma (HCC) is the most common primary malignancy of the liver and the fifth most common malignancy worldwide (Hiraoka et al., 2021). HCC kills about 400,000 people in China each year, accounting for more than 50% of all HCC deaths globally, making it the country’s second leading cause of cancer-related death (Zhang et al., 2021a; Zheng et al., 2021). Despite advances in the clinical management of HCC, the general prognosis of patients with HCC remains extremely poor due to the high rate of metastasis (Wu et al., 2019). RNA methylation is a common RNA modification found in both eukaryotes and prokaryotes. Depending on the different sites of methylation, RNA methylation includes m6A, m5C, m7G, and 2-O-methylation modifications (Zhang et al., 2021b). m7G is a modification in which a methyl group is added to the seventh N-position of RNA guanine (G) (Zhang et al., 2021b). The m7G modification is one of the most common forms of base modification in post-transcriptional regulation, and it is widely distributed in the 5′ cap region of tRNA, rRNA, and eukaryotic mRNA (Guy and Phizicky, 2014; Sloan et al., 2017; Lin et al., 2018). It is important for maintaining RNA processing metabolism, stability, exit from the nucleus, and protein translation (Furuichi et al., 1977; Qin et al., 2020). It is also important for the maintenance of RNA processing and metabolism as well as stability, nucleation, and protein translation (Furuichi et al., 1977). METTL1 catalyzes the m7G modification of tRNAs(Orellana et al., 2021). METTL1 has not been functionally linked to tumorigenesis but is frequently overexpressed and amplified in tumor; it has recently been found to be highly expressed in HCC and associated with poor prognosis (Tian et al., 2019). Previous studies have found that abnormal RNA modifications can influence tumor initiation and progression (Rapino et al., 2018; Delaunay and Frye, 2019; Deb et al., 2020). Although many studies have reported that m6A influences the development of HCC(Qi et al., 2020; Huang et al., 2021a; Xie et al., 2021), the use of m7G as a molecular marker to predict the prognosis of patients with HCC has not been reported before. Because m7G-related genes are still not known to be linked to the prognosis of patients with HCC, there is a strong need to investigate the use of m7G-related genes as molecular markers to predict the prognosis of HCC patients.

## Material and Methods

### Data Acquisition

The Cancer Genome Atlas (TCGA) database (https://tcga-data.nci.nih.gov/tcga/) was used to retrieve publicly available RNA-seq expression data and the corresponding clinical data of 374 HCC samples. The training cohort was the TCGA cohort. Meanwhile, the validation group was created using gene expression data and corresponding clinical data collected from 72 human patients with HCC at the Ninth People’s Hospital of Shanghai Jiaotong University School of Medicine. Before participation in the study, all patients signed a written informed consent form, and the study protocol received ethical approval from the Ninth People’s Hospital of Shanghai Jiaotong University School of Medicine. Age, gender, and TNM stage were among the clinical data collected for the training cohort and validation group (Tables 1, 2). From previous systematic reviews and the MSigDB database, a total of 29 m7G-related genes were extracted (Tomikawa, 2018; Dai et al., 2021; Ma et al., 2021; Zhou et al., 2021).The workflow chart was shown in Supplementary Figure S1. The flowchart was drawn with Figdraw (www.figdraw.com).

**TABLE 1:** Clinical characteristics of the HCC patients used in the derivation cohort.

Characteristic	Levels	Overall
n	—	374
T stage, n (%)	T1	183 (49.3%)
	T2	95 (25.6%)
	T3	80 (21.6%)
	T4	13 (3.5%)
N stage, n (%)	N0	254 (98.4%)
	N1	4 (1.6%)
M stage, n (%)	M0	268 (98.5%)
	M1	4 (1.5%)
Pathologic stage, n (%)	Stage I	173 (49.4%)
	Stage II	87 (24.9%)
	Stage III	85 (24.3%)
	Stage IV	5 (1.4%)
Gender, n (%)	Female	121 (32.4%)
	Male	253 (67.6%)
Age, n (%)	≤60	177 (47.5%)
	>60	196 (52.5%)
Histologic grade, n (%)	G1	55 (14.9%)
	G2	178 (48.2%)
	G3	124 (33.6%)
	G4	12 (3.3%)
OS event, n (%)	Alive	244 (65.2%)
	Dead	130 (34.8%)
Age, median (IQR)	—	61 (52, 69)

**TABLE 2:** Clinical characteristics of the HCC patients used in the validation cohort.

Characteristic	levels	Overall
n	—	72
Age, n (%)	<=60	40 (55%)
	>60	32 (45%)
Gender, n (%)	Female	27 (36%)
	Male	45 (64%)
T stage, n (%)	T1	22 (30%)
	T2	18 (25%)
	T3	18 (25%)
	T4	14 (20%)
N stage, n (%)	N0	68(95%)
	N1	4 (5%)
M stage, n (%)	M0	70(97%)
	M1	2 (3%)
Pathologic stage, n (%)	Stage I	36(50%)
	Stage II	18 (25%)
	Stage III	14 (20%)
	Stage IV	4 (5%)
Histologic grade, n	G1	9(12%)
	G2	35 (48%)
	G3	25 (35%)
	G4	3 (4%)
Age, median (IQR)	—	61.5 (51, 74.25)

### Development and Validation of the m7G-Related Genes Prognostic Model

In the TCGA cohort, the “limma” package in R was used to identify differentially expressed genes (DEGs) between the tumor and adjacent normal tissue, with FDR <0.05 and |log2FC| ≥1. Additionally, m7G-related genes that met the above filtering conditions were considered to be differential. To screen the prognosis of HCC related m7G-related genes, extract survival data of patients with HCC from clinical information files, including survival time and survival status, merge m7G-related genes expression files with survival data, and use of the R software “survival” package coxph function to perform univariate Cox regression analysis. To obtain a generalized linear model and reduce error, the R package glmnet was used to perform 1,000 Cox LASSO regression iterations and 10 cross-validations to obtain a generalized linear model and reduce the error. Further, a multi-factor Cox proportional risk regression analysis was performed to obtain risk genes and construct a risk prognostic model. The disease risk score, which is determined by the parameter β from multivariate Cox proportional risk regression analysis, and the expression of each gene in the sample was used as a predictor of prognosis status in the model. Based on the median risk index, the prognostic model was used to calculate risk scores for the validation and training sets as well as to classify the validation and training sets into high- and low-risk groups. The ability of the regression model to predict survival at 1, 3, and 5 years was assessed using the R software package “survival ROC” to calculate time-dependent subject operating curves (ROC curves). We also evaluate the accuracy of the model using the following statistical metrics: Precision: TP/TP + FP, where TP stands for the number oftrue positive samples and FP stands for the number of falsepositive samples. Accuracy: TP + TN/TP + TN + FP + FN, Recall = TP/TP + FN, where TN is the number of samples that labels and predictions are both negative. FN is the number of samples that labels are positive but predictions are negative. The risk scores obtained were justified by plotting scatter plots and high- low-risk heat maps using survival time and genetic risk models, and validating the value and stability of the regression models in predicting patients’ survival prognosis using a validation set.

### Enrichment Analysis of DEGs and Drug Sensitivity Analysis

Gene Ontology (GO) and Kyoto Encyclopedia of Genes and Genomes (KEGG) pathway analyses were used to assess the functional analysis of m7G-related DEGs. In the form of enrichment analysis, GO covers molecular function (MF), cellular components (CC), and biological process (BP) and provides a comprehensive overview of the functional information of a given gene (Harris et al., 2004). KEGG is a database that integrates genomic, chemical, and systemic functional information and systematically analyzes gene function in terms of genetic and molecular networks to identify functional and metabolic pathways (Du et al., 2014). The Drug Response Gene Signature Database (DSigDB), which integrates drug response microarray data from public databases and scientific literature (Yoo et al., 2015) is accessible through the online enrichment analysis platform Enrichr. Additionally, it also provides drug and target information by creating a DSigDB by screening the top 500 upregulated and downregulated genes for drug signatures. Over 1,300 drugs, 7,000 microarrays, and 800 targets are currently in the DSigDB, enabling computational drug repositioning to develop novel drugs targeting core genes (Federer et al., 2016). In this study, DSigDB was used to screen drug candidates that interact with DEGs to provide a reference for disease-specific therapy. *p* < 0.05 was considered as a statistically significant difference.

### Analysis of Immune Infiltration

Infiltration of the immune system has been associated with cancer survival and progression (Wu et al., 2021). We assessed the correlation between risk score and immune infiltration level. The R package “GSVA” was used to quantify the infiltration of each immune cell type in the HCC using a single-sample gene set enrichment analysis (ssGSEA) algorithm.

### Quantitative Real-Time PCR

Tumor tissue and normal tissue specimens (>4 cm from the tumor specimen) were collected from 72 HCC patients admitted to the Ninth People’s Hospital of Shanghai Jiaotong University School of Medicine, and all the specimens were diagnosed pathologically. Total RNA was extracted using the Trizol method, and the tested RNA was subjected to reverse transcription and PCR reactions according to the instructions on the reverse transcription kit (Takara, batch: AJ60796A) and PCR reagent (Roche, batch: 41472600). The relative expression levels of mRNA were calculated using the 2^-ΔΔCt method (ΔCt = Ct (target gene-internal reference gene); ΔΔΔCt = ΔCt–Δctmin), and the paired t-test was used to compare the differences in gene expression in tumor tissues and normal paracancerous tissues. *p* < 0.05 was considered statistically significant. The primers used in this study were obtained from TsingKe biological technology (Nanjing, China). The primers used are as follows: CYFIP1 (forward 5′- TCC​CCA​TTG​AGA​TGT​CGA​TGC -3′, reverse 5′- ACT​GCT​TGT​TGA​ACC​TGG​TGA -3′), EIF4E2 (forward 5′- ACA​ACA​AGT​TCG​ACG​CTT​TGA -3′, reverse 5′- TCT​CTT​GCT​ACT​GCT​CTG​ATT​CT -3′), EIF4E2 (forward 5′- ACA​ACA​AGT​TCG​ACG​CTT​TGA -3′, reverse 5′- TCT​CTT​GCT​ACT​GCT​CTG​ATT​CT -3′), EIF4G3 (forward 5′- CCT​AGA​GCT​ACC​ATC​CCG​AAC -3′, reverse 5′- GGG​CCA​CTA​TGA​CGG​TAC​TG -3′), GEMIN5 (forward 5′- CCT​CCG​TCT​TCC​TTG​TCC​G -3′, reverse 5′- CAG​AGA​CCC​TTT​CGG​TGT​GTC -3′), NCBP2 (forward 5′- AAA​ACG​CCA​TGC​GGT​ACA​TAA -3′, reverse 5′- GCC​TGC​CCT​CCT​TAA​AGC​C -3′), NUDT10 (forward 5′- CGG​TCC​GAG​AGG​TGT​ACG​A -3′, reverse 5′- AAT​CTT​CCC​AAT​CCT​CCA​GCA -3′), WDR4 (forward 5′- CCA​CCT​CCA​TAG​CAA​GCA​GTG -3′, reverse 5′- ACG​CTT​ACT​GTC​ATC​GGT​TAA​AG -3′).

## Results

### Identification of Prognostic m7G-Related DEGs and Construction of a Prognostic Model

**FIGURE 1:**
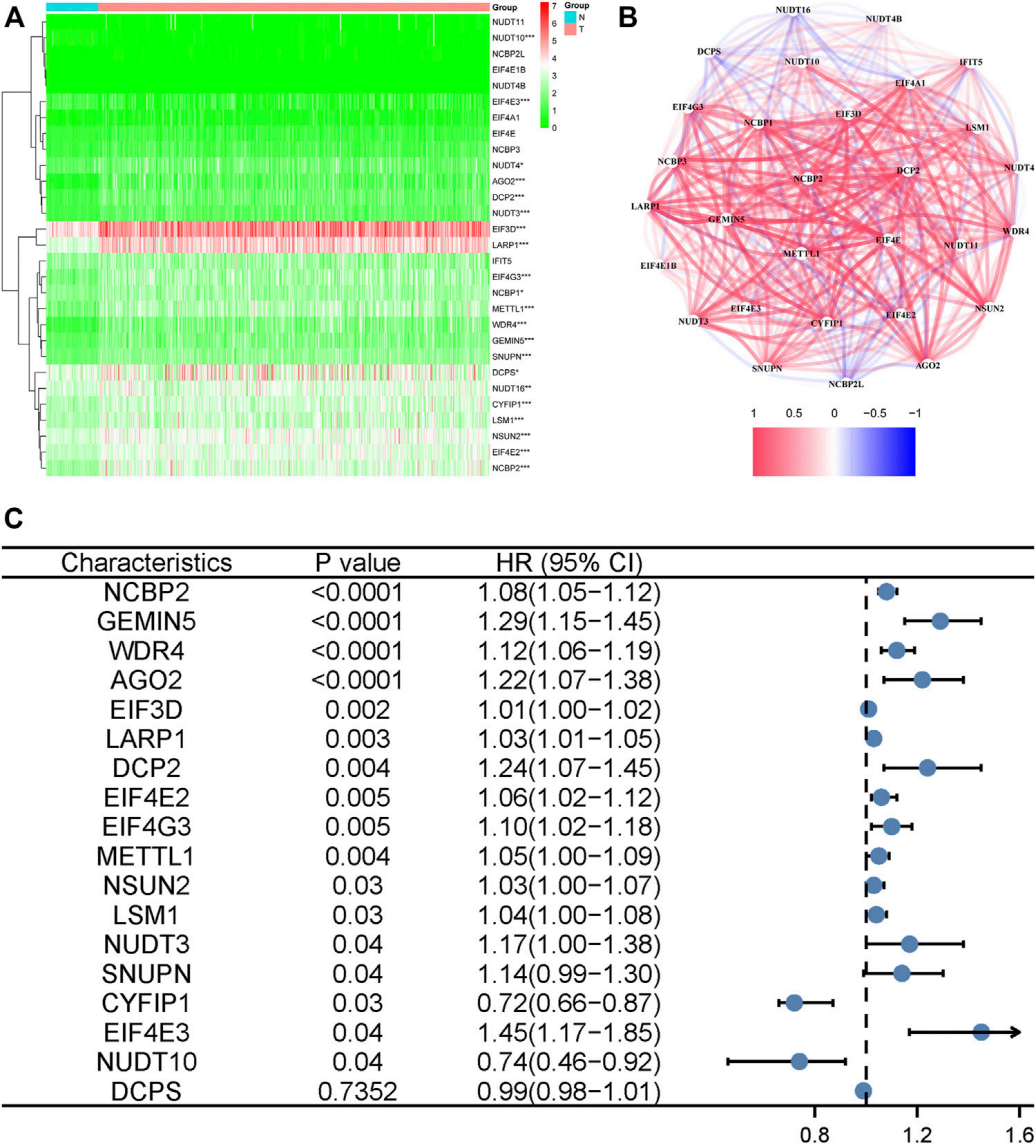
Identification of the candidate genes. **(A)** Heatmap of the differentially expressed genes in the two groups. *** denotes *p* < 0.001, ** denotes *p* < 0.01, and * denotes *p* < 0.05. **(B)** The relationship between necroptosis-related differentially expressed genes. A positive correlation is indicated by red, while a negative correlation is indicated by blue. **(C)** Forest plots showing the results of a univariate Cox regression analysis between gene expression and overall survival.

**FIGURE 2:**
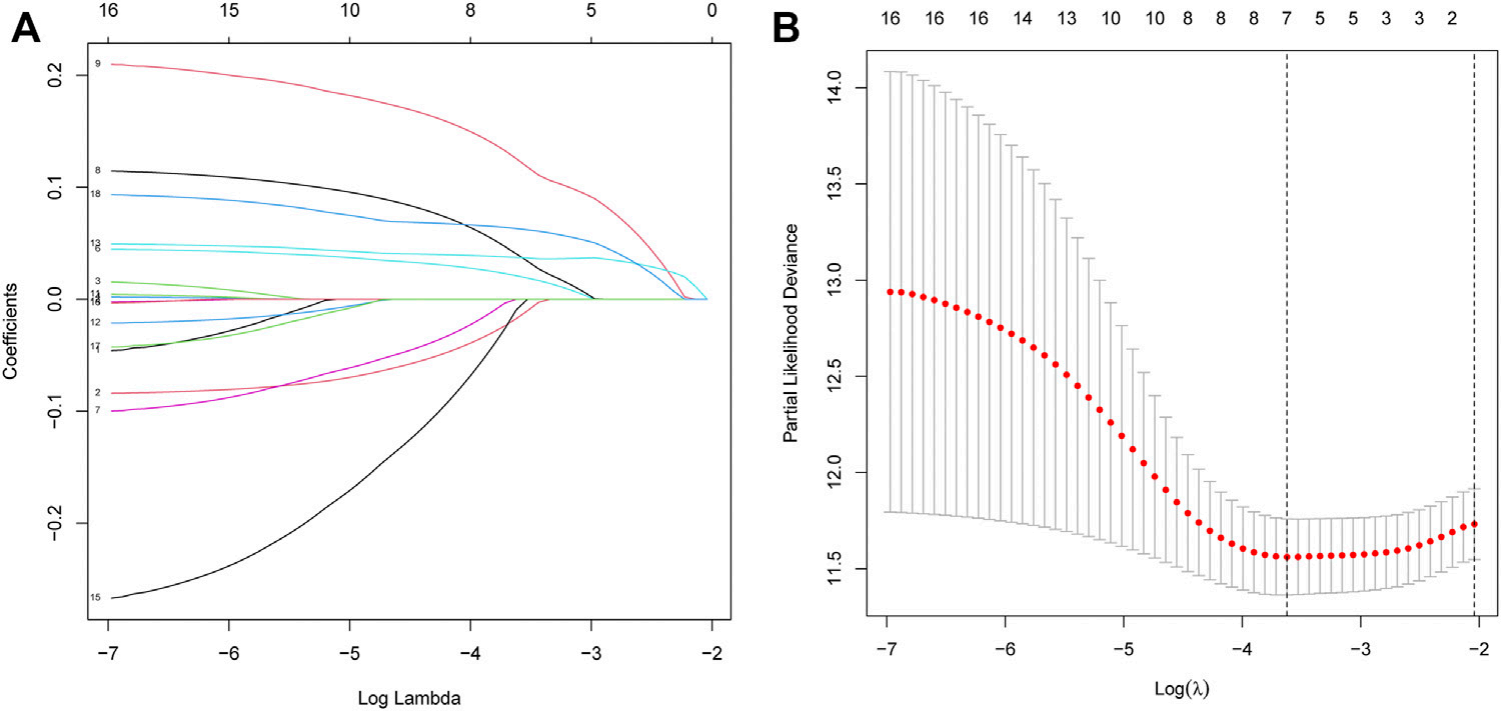
Processes of LASSO Cox model fitting. **(A)**. The log(lambda) sequence was used to plot the profile of coefficients in the model at varying levels of penalization. **(B)**. Cross-validated error tenfold (the first vertical line equals the minimum error, whereas the second vertical line shows the cross-validated error within a minimum of 1 standard error).

**FIGURE 3:**
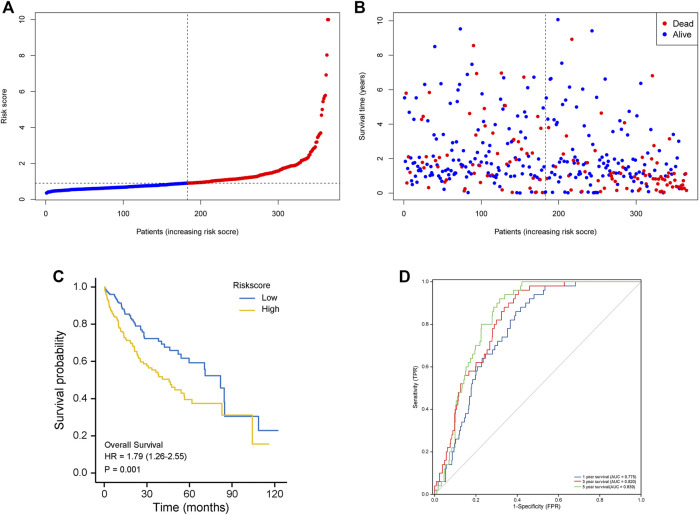
The seven m7G-related genes signature model’s prognostic value in the test (TCGA) cohort. **(A)** The risk score distribution and median values. **(B)** overall survival status, overall survival, and risk score distributions in the derivation cohort. **(C)** Kaplan–Meier curves show the overall survival of patients in the high-risk and low-risk groups. **(D)** The area under the curves of time-dependent ROC curves shows the risk score’s prognostic performance.

Fifty normal tissue samples and 374 tumor tissue samples of HCC were obtained from the TCGA database. Most m7G-related genes were found to be differentially expressed between the cancer and paracancerous tissues (19/29, 65.5%, Figure 1A green: low expression level; red: high expression level). The relationship between the m7G-related DEGs is shown in Figure 1B. In the univariate regression analysis, 17 DEGs were associated with overall survival (OS) prognosis (Figure 1C), and Lasso regression analysis identified seven key genes (Figures 2A,B). Therefore, we selected the seven genes mentioned above to construct a multifactorial Cox regression model and calculated risk scores. The risk score was calculated using the following formula: -0.09187 * expression level of *CYFIP1* + 0.04210 * expression level of *EIF4E2* + 0.10574 * expression level of *EIF4G3* + 0.20427 * expression level of *GEMIN5* + 0.04164 * expression level of *NCBP2* −0.26358 * expression level of *NUDT10* + 0.07544 * expression level of *WDR4*. According to the median scores, patients were divided into high- and low-risk groups (Figure 3A). Patients in the high-risk group had a higher chance of dying than patients in the low-risk group (Figure 3B). When survival analysis was performed, the Kaplan–Meier curve showed that the low-risk group had a significantly higher survival rate than the high-risk group, with *p* < 0.005 (Figure 3C). The predictive performance of the OS risk score was assessed using the ROC curve, the area under the curve (AUC) of the prognostic risk assessment model for the seven m7G-related genes was 0.775, 0.820, and 0.839 at 1, 3, and 5 years, respectively (Figure 3D). Supplementary Table S1 shows the results of the precision, recall and accuracy metric of the model.

### Validation of Prognostic Models

**FIGURE 4:**
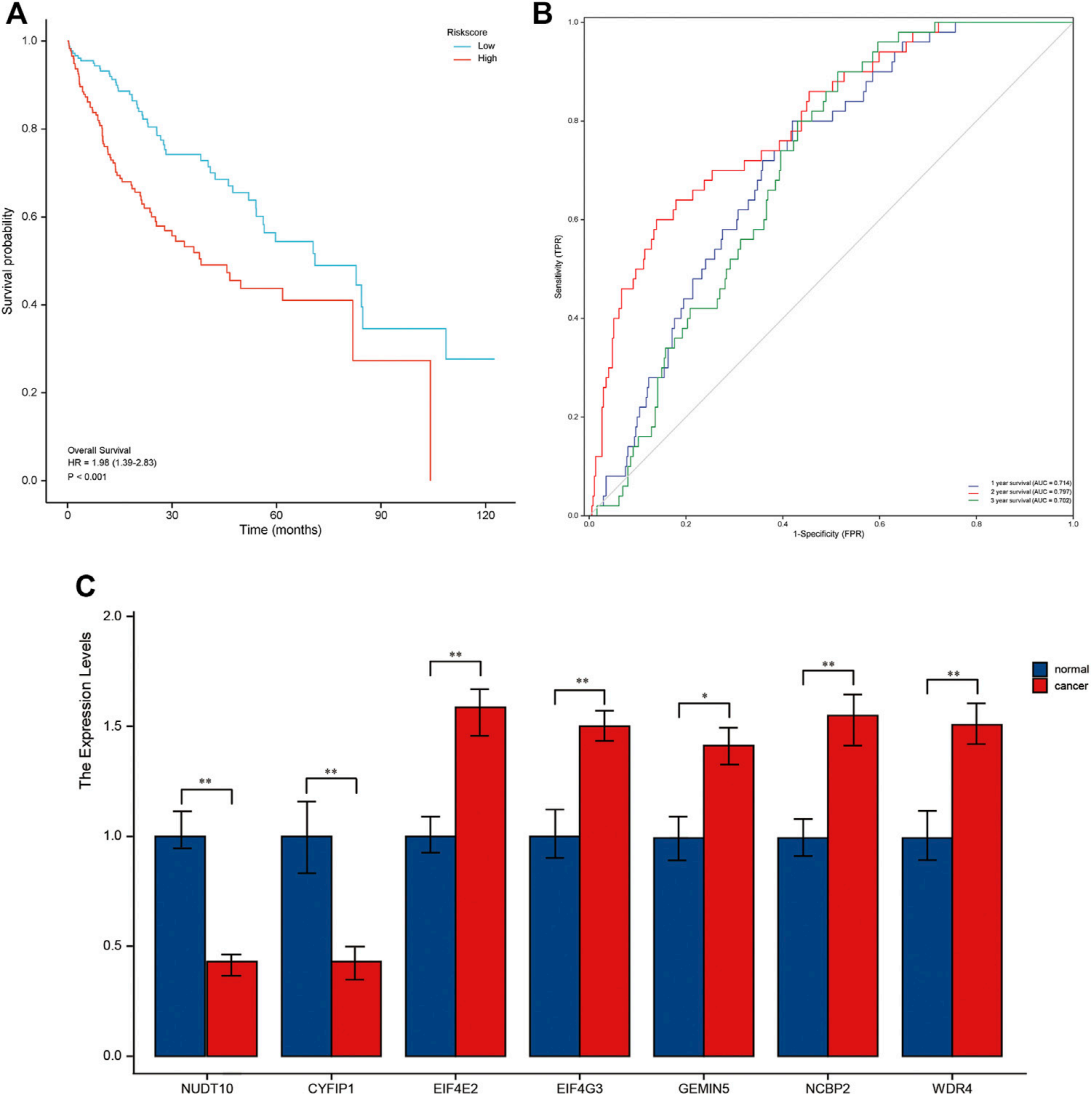
A validation cohort was used to validate the seven m7G-related genes. **(A)**. Kaplan–Meier curves showing overall survival of patients in the high-risk and low-risk groups. **(B)**. The area under the curves of time-dependent ROC curves indicates the risk score’s prognostic performance. **(C)**. Results of quantitative real-time PCR analysis.

Patients in the validation cohort were calculated using the same risk score formula as the TCGA cohort, and the median of the calculation was divided into a high-risk group and a low-risk group to validate the robustness of the prognostic model built for the TCGA cohort. The high-risk group had a worse prognosis than the low-risk group, similar to the TCGA findings (Figure 4A). Furthermore, the AUC of the 7-genes signature was 0.869, 0.904, and 0.808 at 1, 3, and 5 years, respectively (Figure 4B). *NUDT10, CYFIP1* were highly expressed in normal tissues by qRT-PCR, and the remaining five genes were highly expressed in the validation group tumors (Figure 4C). Supplementary Table S1 shows the results of the precision, recall and accuracy metric of the model in validation set.

### Independent Prognostic Value of the Risk Model

The prognostic model was subjected to Cox univariate and multifactorial analyses to see if the 7-gene marker model was independent of other clinical variables. The 7-genes marker model was found to be an independent factor affecting OS in patients with HCC in both univariate and multifactorial Cox regression analyses (*p* < 0.05, Table3).

**TABLE 3:** The model was found to be an independent factor affecting OS in patients with HCC in both univariate and multifactorial Cox regression analyses.

Characteristics	Univariate Analysis		Multivariate Analysis	
	Hazard Ratio (95% CI)	*p* Value	Hazard Ratio (95% CI)	*p* Value
T stage				
T1&T2	Reference	0.3	—	—
T4&T3	1.27 (1.18–1.45)			
N stage				
N0	Reference	0.2	—	
N1	1.52 (1.32–1.91)			
M stage				
M0	Reference	0.93	—	—
M1	1.86 (1.41–1.91)			
Age				
≤65	Reference	**<0.001**	2.86 (1.38–4.96)	**0.04**
>65	4.81 (2.48–5.86)			
Pathologic stage				
Stage I&Stage II	Reference	**0.03**	1.72 (0.46–2.71)	0.54
Stage III&Stage IV	1.32 (1.16–1.71)			
Riskscore				
Low	Reference	**<0.001**	2.86 (1.38–4.96)	**<0.001**
High	2.59 (1.73–4.05)			

Values in bold are significant correlations (P < 0.05)

### Enrichment Analysis

**FIGURE 5:**
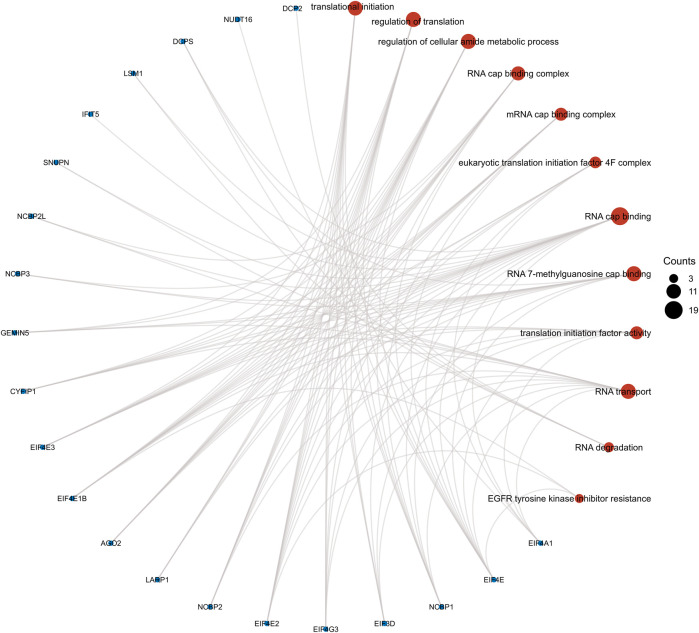
Results from gene ontology functional and Kyoto Encyclopedia of Genes and Genomes pathway enrichment analyses for the identified differentially expressed genes.

**FIGURE 6:**
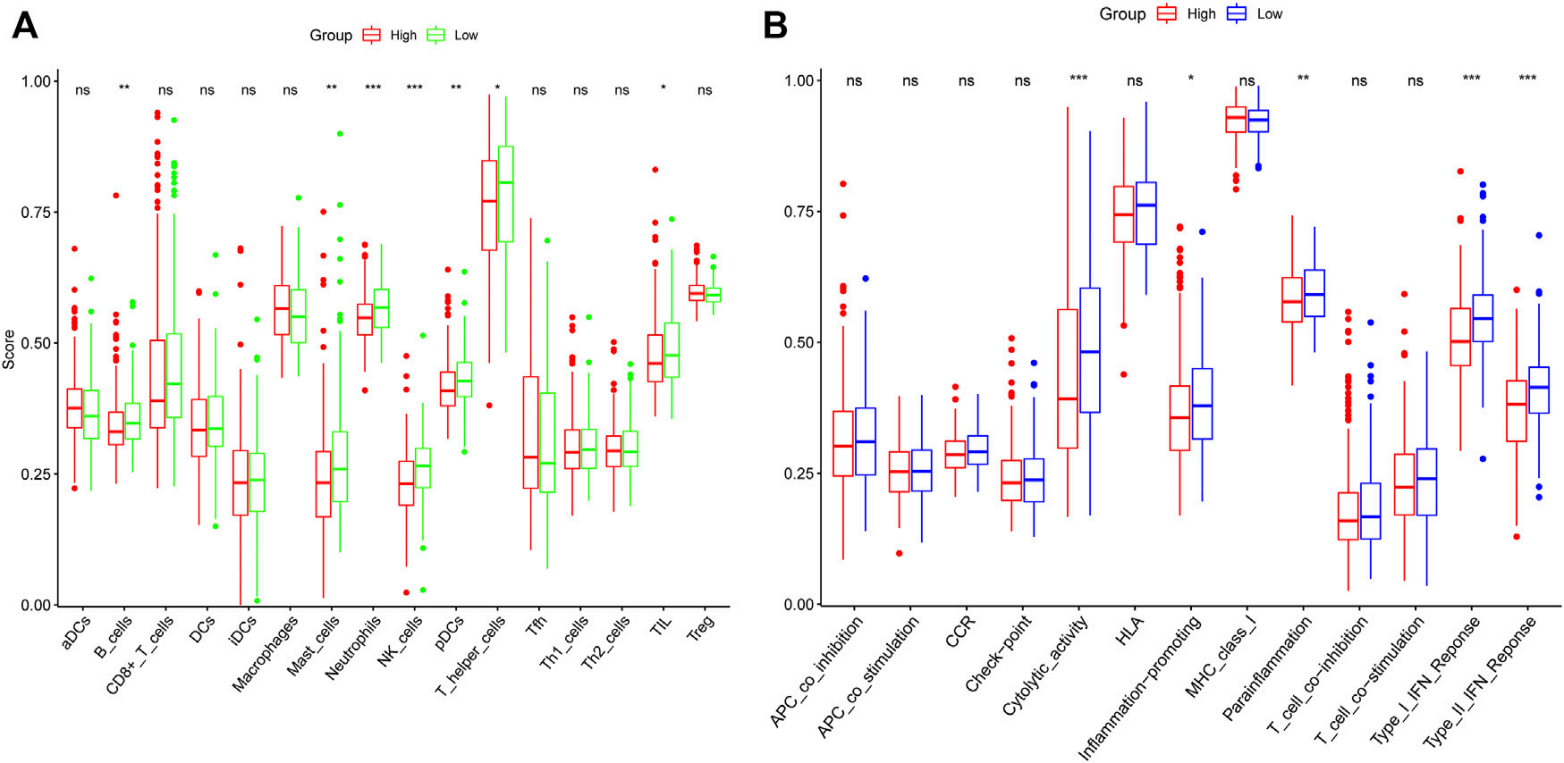
The single-sample gene set enrichment analysis scores of different risk groups in the derivation cohort were compared. The scores of 16 immune cells **(A)** and 13 immune-related functions **(B)** are displayed in boxplots.

We performed GO enrichment analysis of m7G-related DEGs and found that BP focusing on translational initiation, regulation of translation, regulation of cellular amide metabolic process, and CC mainly enriched in RNA cap-binding complex, mRNA cap-binding complex, eukaryotic translation initiation factor 4F complex, and MF focusing on RNA cap binding, RNA 7-methylguanosine cap binding, and translation initiation factor activity. KEGG analysis showed that RNA transport, RNA degradation, EGFR tyrosine kinase inhibitor resistance, the longevity regulating pathway were all significantly activated (Figure 5). The results of ssGSEA showed the low-risk group had higher levels of B cells, mast cells, neutrophils, NK cells, pDCs, T helper cells, and TIL infiltration than the high-risk group (Figure 6A). Meanwhile, in the low-risk group, immune-related functions such as cytolytic activity, inflammation-promoting, parainflammation, type II IFN response, and type I IFN response were more active (Figure 6B). The above results could also explain why patients in the low-risk group have a better moral prognosis. When the seven genes were subjected to multifactorial Cox analysis, the *CYFIP1, EIF4G3,* and *GEMIN5* were statistically significant, implying that they could be independent prognostic factors for HCC.

### Drug Sensitivity Analysis

Based on DSigDB analysis, this study identified 10 sensitivity drugs (Supplementary Table S2). Trichostatin A (mechanism of oncogenic effects *via* miR-34-mediated pathways (Song et al., 2015)) had the highest negative correlation score. This suggests that it has a potential therapeutic effect in HCC. Vorinostat is a histone deacetylase inhibitor that is effective against HCC in preclinical studies (Hsu et al., 2014). Cimetidine’s ability to scavenge free radicals, increase the expression and activity of antioxidant enzymes, regulate the body’s immune function and protect hematopoietic function may explain why it can inhibit the growth and metastasis of many experimental tumors and improve the survival rate of patients with tumors (Sirota et al., 2011; Massari et al., 2020).

## Discussion

The process of transferring methyl groups from one reactive methyl compound to another is known as methylation. RNA methylation, DNA methylation, histone modification, non-coding RNA modification, and chromatin rearrangement are common epigenetic modifications (Huang et al., 2021b). A methyl group is added to position N7 of riboguanosine to produce m7G (Cowling, 2009). This capped mRNA modification is facilitated by RNA guanine-7 methyltransferase and regulates gene expression, mRNA splicing, transcription, and nuclear export of mRNA, as well as regulates mRNA translation (Enroth et al., 2019). Recently, RNA methylation has been associated with a variety of human physiologies and diseases, particularly with tumor immunity (Zhang et al., 2021b). For example, impaired m7G tRNA modification leads to different forms of microcephalic primordial dwarfism and Galloway-Mowat syndrome (Dai et al., 2021). Dai demonstrates the critical function of METTL1-mediated m7G tRNA modification in promoting ICC intrahepatic cholangiocarcinoma *in vivo* (Dai et al., 2021). In addition to this, impaired m7G tRNA modification has been shown to be associated with glioblastoma multiforme (GBM), liposarcoma (LPS), melanoma and acute myelogenous leukaemia (AML) (Orellana et al., 2021). However, the relationship between m7G and HCC prognosis is currently unclear.

In this study, we explored the relationship between m7G-related genes and the prognosis of HCC patients. Using a prognostic model of seven m7G-related genes (*CYFIP1, EIF4E2, EIF4G3, GEMIN5, NCBP2, NUDT10,* and *WDR4*), we predicted the prognosis of patients with HCC. The KM curve and accuracy of the model were used to verify the model’s validity. Additionally, the model was also combined with clinical information on HCC to analyze and screen factors affecting the prognosis of HCC. The findings revealed that a high-risk score is an independent risk factor affecting the prognosis of patients with HCC. *CYFIP1*, a newly discovered tumor suppressor gene, shows some tumor-suppressive effects in breast, lung, colon, and bladder cancers (Silva et al., 2009; Teng et al., 2016a; Teng et al., 2016b). It is also associated with tumor metastasis (Chen et al., 2016). We found that *CYFIP1* is an oncogene in HCC, and its expression in the tissues of HCC patients was higher than in normal tissues in the vicinity of the tumor. *GEMIN5* can regulate translation and has been reported to specifically bind to the m7G cap (Xu et al., 2016). We found that the expression of *GEMIN5* was higher in the tissues of patients with HCC than in normal tissues adjacent to cancer and that patients with high expression had a poor prognosis. In ovarian carcinoma, *NCBP2* has been reported as a key target gene (Wei et al., 2015). In contrast, *NCBP2* was a risk factor for patient prognosis in patients with HCC. *WDR4* is highly expressed in fetal heart, kidney, and brain tissues and it is important for development (Abedini et al., 2018).

The tumor microenvironment plays a crucial role in immune response suppression or enhancement (Li et al., 2017). The microenvironment of patients with HCC contains a large number of inflammatory and immune factors, and the differential expression of inflammatory and immune cell numbers and phenotypes correlate with the prognosis of patients with HCC. Immunity modulation may be an effective means of treating tumors (Wu et al., 2012). We performed an immune cell infiltration analysis to assess the relationship between risk scores and overall survival of patients in the context of immune system response. Our results suggest that immune cell infiltration levels in low-risk patients were remarkably higher than those in high-risk patients. T cells are at the center of immunotherapy for tumors. Mobilization of immune cells to kill tumor cells is the most effective and safe treatment method. In recent studies, T cells have ben shown to be closely related to tumor immune escape mechanisms and can be used as a prognosis indicator to some extent (Okita et al., 2015; Hirano, 2018). Treg cells are a subset of T cells that regulates the body’s autoimmune response by secreting anti-inflammatory cytokines, expressing FoxP3 specifically, and exerting immunosuppressive effects through interleukin-10 (IL-10), and promoting transforming growth factor-alpha (TGF-α). Treg cell numbers were found to be negatively correlated with OS in patients with tumors, and the prognostic role of Treg cells was correlated with tumor cell subtype and tumor stage (Litwin et al., 2021). Drug sensitivity testing revealed potential drugs that could regulate m7G-related genes. Notably, drugs like trichostatin A, vorinostat, and rifabutin were found to have a negative correlation with the expression of these m7G-related genes. This provides a new drug treatment option for HCC.

## Conclusion

In conclusion, this study developed a risk prediction model by analyzing m7G-related genes. The model has good prediction accuracy and can be used to divide patients with HCC into high- and low-risk groups, with the high-risk group being an independent risk factor for the prognosis of HCC. Moreover, we also looked at the relationship between risk scores and immune activities.

## Data Availability

The original contributions presented in the study are included in the article/Supplementary Material, further inquiries can be directed to the corresponding author.

## Supplementary Material

Supplementary Table S1 shows the results of the precision, recall and accuracy metric of the model.

Supplementary Table S2 The top 10 results from the drug sensitivity analysis.
