# Supplementary material for: m7G Methylation-Related Genes as Biomarkers for Predicting Overall Survival Outcomes for Hepatocellular Carcinoma
Source: Front Bioeng Biotechnol. 2022 May 10;10:849756. doi: 10.3389/fbioe.2022.849756 (PMC9127183; doi:10.3389/fbioe.2022.849756)
Supplement: Supplementary file 1 [file Table1.docx]

|  | **Precision** | **Recall** | **Accuracy** |
| --- | --- | --- | --- |
| **derivation cohort** |  |  |  |
| 1 year | 0.835 | 0.822 | 0.840 |
| 3 year | 0.840 | 0.837 | 0.832 |
| 5 year | 0.837 | 0.844 | 0.801 |
| **validation cohort** |  |  |  |
| 1 year | 0.782 | 0.755 | 0.739 |
| 3 year | 0.756 | 0.726 | 0.724 |
| 5 year | 0.721 | 0.703 | 0.717 |
