# Supplementary material for: m7G Methylation-Related Genes as Biomarkers for Predicting Overall Survival Outcomes for Hepatocellular Carcinoma
Source: Front Bioeng Biotechnol. 2022 May 10;10:849756. doi: 10.3389/fbioe.2022.849756 (PMC9127183; doi:10.3389/fbioe.2022.849756)
Supplement: Supplementary file 2 [file Table2.docx]

| Term | P-value |
| --- | --- |
| trichostatin A HL60 DOWN | 9.25E-05 |
| vorinostat HL60 DOWN | 1.15E-04 |
| rifabutin MCF7 DOWN | 1.28E-04 |
| cimetidine PC3 DOWN | 1.30E-04 |
| clopamide HL60 DOWN | 2.20E-04 |
| chlortetracycline HL60 DOWN | 2.22E-04 |
| 2-Methylcholine CTD 00002006 | 0.002557 |
| azacyclonol HL60 DOWN | 0.059916 |
| METHYL METHANESULFONATE CTD | 0.00331 |
